# Successful Extraction of the Child by the Cœsarean Section, after the Death of the Mother

**Published:** 1850-12

**Authors:** 


*Successful extraction of the Child by the Cæsarean Section, after the Death of the Mother.* By George Harley, Esq., House Surgeon in the Edinburgh Maternity Hospital.

Catharine Davidson, aged 39, unmarried, was admitted into the Royal Maternity Hospital, Edinburgh, in the beginning of March, 1850, being in the seventh month of her second pregnancy, and complained of shortness of breathing in ascending stairs, and of symptoms of dyspepsia. Had menstruation last on 12th August, 1849, and expected to be delivered about the middle of May.

Being much troubled with swelled legs, and her face having a puffy, unusually pale, appearance, her urine was tested ten days before her death, and showed no appearance of albumen on the application of heat or nitric acid, separately and combined.

Thursday, 25th April, 1850.—About 25 minutes to 11, a. m., I was summoned to the kitchen by the matron, as one of the patients was in danger of suffocation. My first inquiry was, "had she lately taken any food?" The answer was in the negative. On reaching the kitchen, I found Catharine Davidson sitting on a chair, in the washing house, close to the kitchen door, supported by two of the other patients, with eyes prominent, lips becoming blue, respiration very hurried, with great effort and heaving of the chest; her countenance altogether denoting the most intense anxiety. All that she could say was, "I am gone! O, doctor, save me!" Hearing a distinct half crowing, half gurgling sound in her throat, I immediately ordered her dress to be loosened, and her stays torn off. She was now spitting up a brownish, glairy, semi-fluid matter.

We supported her, and got her up stairs to ward No. 4, having to stand still on the way several times, to allow her to recover her breath. As soon as she sat down in bed, I ran and prepared an emetic of ℥ss. sulphate of zinc, which she willingly swallowed. Her symptoms gradually, though quickly, becoming more urgent, I proposed performing laryngotomy; she laid herself upon her back, and the operation was quickly completed. On bringing her head forwards over the bed, white frothy mucus ran out of her mouth, and some from the wound. There not being a tracheotomy tube in the house, I cut about three and a half inches off a full-sized flexible male catheter, and inserted it into the wound; but finding the mucus did not come readily through it, it was immediately withdrawn. I then repeated the emetic, as the former dose seemed greatly to have assisted expectoration, and in less than five minutes above eight ounces of white frothy mucus were collected in a vessel. The patient now became quite livid in the face, her eyes seemed starting from their sockets; and after tossing about her arms, and giving one or two gasping inspirations, she fell backwards insensible. The symptoms still simulating those of spasmodic closure of the glottis, or some other obstruction at the larynx, and thinking that the artificial opening was not large enough to admit sufficient air to carry on the respiration, or allow her to expectorate freely, we pulled the bed to the nearest window, and I immediately proceeded to perform tracheotomy; but before the operation was much more than half finished, all attempts at respiration ceased.

The matron, a most intelligent person, having had her hand on the pulse, watching its decline, told me that it had ceased to be perceptible for some minutes. On touching the wrist, I found she was perfectly correct; and, on putting my ear to the chest, I heard no pulsation at the heart. A single glance at the patient's face showed evidently that she was dead; the frothy mucus was running out at the mouth and nostrils, the eyes were fixed, and the pupils dilated.

All hope of saving the woman being lost, my next thought was to save the life of the child; so I again snatched up the bistoury, ripped down the patient's dress, and instantly made an incision in the mesial line, through the parietes of the abdomen, commencing a little above the symphysis pubes, keeping close to the right side of the umbilicus, and terminating a little above it. The uterus then appeared, and I proceeded to make incisions in it, to avoid wounding the child. In making these incisions none of the intestines came in the way of the knife, and there was very little or no bleeding from the wounds.

When the cavity of the uterus was reached, the liquor amnii escaped. I put the two first fingers of my left hand into it, laid the back of the knife against them, and cut downwards and outwards. One side of the nates now appeared. I then put the right hand into the uterus, caught hold of the first thing that came in the way, which happened to be a leg, and withdrew the child without any difficulty, the uterus not contracting round the neck.

The child, on extraction, looked beautiful and clean, as if it had been carefully washed; it was to all appearance quite dead, no pulsation being felt either at heart or cord. I dashed cold water on its chest, gave it a rub, and then put my mouth to its mouth, depressed and pushed back the larynx, held the nostrils with the one hand, and pressed on the chest with the other, after each time that I filled the child's lungs with air.

After a few minutes I stopped to take breath, and during that time I applied friction and aqua ammoniæ to the breast; and on using artificial respiration for some minutes more, the child's heart began to beat, and the pulsations in the cord became distinctly visible; a legature having been put upon it, the child was cut away. It was a male, weighing 6 lbs. 12 ozs. and measuring 18½ inches. Shortly afterwards I put my hand into the uterus, and peeled the placenta from the back part and right side of the organ, as it would not come away by pulling at the cord; it weighed 1 lb. 4 ozs., and the cord measured 20 inches. The wound was stitched up, and the body removed into the delivery room, where the post-mortem examination took place.

Not more than twenty minutes elapsed from the time the patient was seized in the kitchen till all was over.

*Autopsy, twenty-four Hours after Death.* Present—Dr. Thompson; Mr. Rolston, surgeon; and Mr. Harley, house-surgeon.

*External Surface.*—Countenance livid, lips bluish-purple, eyes prominent, pupils dilated; an incision 8½ inches long, extending from a little above the symphysis pubis, keeping to the right side of the umbilicus, and terminating about an inch above it.

*Pulmonary Organs.*—Lungs pale and white at the margins, which were emphysematous, touching each other opposite the 2d and 3d ribs; six ounces of serum in the left pleura, as much, if not more, in the right, which was not measured, as some blood from the incisions made in opening the chest, had mingled with it.

On incision being made into the lungs, they were seen to be quite full of mucus, which exuded on presure; the crepitation was very slight, in consequence of the œdema; a few hard substances like tubercles were found throughout their substance.

*Cardiac Organs.*—On opening the pericardium, four ounces of serum were found in it; it was otherwise healthy. Heart rather pale, weighing 14 ounces. Ventricles contracted, without any blood in their cavities; left auricle dilated; mitral valves cartilaginous, and scarcely admitting the point of the fore-finger. The corpora aurantii on the aortic valves were very much hypertrophied, preventing complete closure of the aortic orifice, and consequently permitting regurgitation. Right side of heart natural.

*Lrynx and Trachea* both quite healthy; no vestige of any lesion, except the laryngotomy wound; epiglottis and vocal cords healthy.

*Abdomen.*—*Kidneys* full size and quite healthy. *Spleen* healthy and of usual size. *Liver* not examined. *Intestinal canal* quite normal. *Uterus* not contracted, the wound in its anterior surface quite distinct. Some urine being drawn off by the catheter, was found to be slightly coagulable by nitric acid, but not by heat, till nitric acid was also added. It was not changed by caustic potash and heat.

*Examination of Head, twenty-nine Hours nfter Death.*—Present—Dr. Keiller and Mr. Harley.

*Skull* thick, with dura mater adherent to it; no extravasation external to dura mater. Superior longitudinal sinus empty. On raising the dura mater, a quantity of serous effusion was found beneath the arachnoid membrane, and a large quantity flowed also from the spinal canal. *Brain* quite healthy, rather anænic than otherwise; arteries empty, and choroid plexuses very anemic; ventricles containing rather more than the usual amount of serum. Nothing could be detected in the brain to account for death.*

From information subsequently received from the relations, it was found that, when 14 or 15 years of age, she had been confined to bed for a fortnight or three weeks with an attack of acute rheumatism. Four years ago she had had a severe cold, and had always complained more or less of colds since, together with shortness of breath on going up stairs, and occasional slight palpitation; during the past winter her cough had been more troublesome than usual.—*Edinburgh Monthly Jour. of Medical Science.*

---

*Serous effusion in this arachnoid was probably the immediate cause of death. See Original Communications, Art. No. III.—*Editor Buffalo Journal.*

